# Predicting enhancer-promoter interaction based on epigenomic signals

**DOI:** 10.3389/fgene.2023.1133775

**Published:** 2023-04-18

**Authors:** Leqiong Zheng, Li Liu, Wen Zhu, Yijie Ding, Fangxiang Wu

**Affiliations:** ^1^ School of Mathematics and Statistics, Hainan Normal University, Haikou, China; ^2^ Yangtze Delta Region Institute (Quzhou), University of Electronic Science and Technology of China, Quzhou, China; ^3^ Key Laboratory of Computational Science and Application of Hainan Province, Haikou, China

**Keywords:** enhancer-promoter interaction, machine learning, ChIA-PET, random forest, epigenomic signals

## Abstract

**Introduction:** The physical interactions between enhancers and promoters are often involved in gene transcriptional regulation. High tissue-specific enhancer-promoter interactions (EPIs) are responsible for the differential expression of genes. Experimental methods are time-consuming and labor-intensive in measuring EPIs. An alternative approach, machine learning, has been widely used to predict EPIs. However, most existing machine learning methods require a large number of functional genomic and epigenomic features as input, which limits the application to different cell lines.

**Methods:** In this paper, we developed a random forest model, HARD (H3K27ac, ATAC-seq, RAD21, and Distance), to predict EPI using only four types of features.

**Results:** Independent tests on a benchmark dataset showed that HARD outperforms other models with the fewest features.

**Discussion:** Our results revealed that chromatin accessibility and the binding of cohesin are important for cell-line-specific EPIs. Furthermore, we trained the HARD model in the GM12878 cell line and performed testing in the HeLa cell line. The cross-cell-lines prediction also performs well, suggesting it has the potential to be applied to other cell lines.

## 1 Introduction

Enhancers and promoters are two of the most critical regulatory elements of gene transcription in the eukaryotic genome (Maston et al., 2006). The physical interactions between them precisely regulate spatiotemporal gene expression, which contributes to complex cellular functions. Aberrant connections between enhancers and promoters may lead to abnormal expression of disease-related genes (Krijger and De Laat, 2016). Therefore, the study of how enhancers and promoters interact can improve our understanding of health and disease. The primary mechanism of enhancer-promoter interaction is chromatin looping (Rubtsov et al., 2006; Miele and Dekker, 2008), which allows distal enhancers to contact the target gene promoters in three-dimensional space (Lv et al., 2021). Such long-range regulatory interactions play a significant role in tissue-specific gene expression (Maston et al., 2006; De Laat and Duboule, 2013) and can link the regulatory element to the target gene (Corradin et al., 2014). In recent decades, the identification of EPIs has relied on high-throughput experimental techniques, such as chromosome conformation capture (3C) (Dekker et al., 2002), 4C (Splinter et al., 2012), 5C (Sanyal et al., 2012), Hi-C (Lieberman-Aiden et al., 2009), Hi-C capture (Schoenfelder et al., 2015), DNase-Hi-C (Ma et al., 2015), and ChIA-PET (Li et al., 2012; Heidari et al., 2014). These experimental approaches are effective in identifying EPIs but are time-consuming and expensive (Ecker et al., 2012). Thus, a more cost-effective method is required for predicting enhancer-promoter interactions. To address this problem, machine learning methods are used to predict EPIs by using available genomic or epigenomic data.

Many deep learning methods have been proposed for predicting EPIs based on DNA sequence, including SPEID, SIMCNN, and EPIVAN. SPEID (Singh et al., 2019) and SIMCNN (Zhuang et al., 2019) employ CNN-based approaches, while EPIVAN (Hong et al., 2020) incorporates an attention mechanism for improved prediction accuracy. Although they achieved good results using only DNA sequences, the cell-line-specific nature of EPIs (Heidari et al., 2014; Ma et al., 2015) presents a challenge (Lv et al., 2021; Ao et al., 2022a). For instance, the same pair of enhancer and promoter contacts in some cell lines, but not in others, despite the DNA sequences have not changed (Schöler and Gruss, 1984). To address this issue, several models have been developed to identify cell-line-specific EPIs using epigenomic signals, including chromatin accessibility, the binding of special transcription factors, and histone modification levels. For example, RIPPLE (Roy et al., 2015) provides a systematic approach for predicting and interpreting EPIs in a cell-line-specific manner using a variety of epigenomic features. However, many epigenomic signals are not available for all cell lines.

Based on the aforementioned analyses, we considered using as few epigenomic features as possible to build machine learning models to predict cell-line-specific EPIs. Loose chromatin is a prerequisite for loop formation. The H3K27ac ChIP-seq and ATAC-seq data are often used to represent chromatin accessibility. Chromatin interaction decays with distance. RAD21 is a subunit of cohesin that play important role in a loop formation. Therefore, the four types of features were extracted to train the models. By comparing several machine learning classifiers, the random forest was selected due to the high accuracy. Finally, we compared our HARD model with the sequence-based and other epigenomic features-based models. The results showed that our model outperformed them both in the same cell line and cross-cell-lines.

## 2 Materials and methods

**FIGURE 1:**
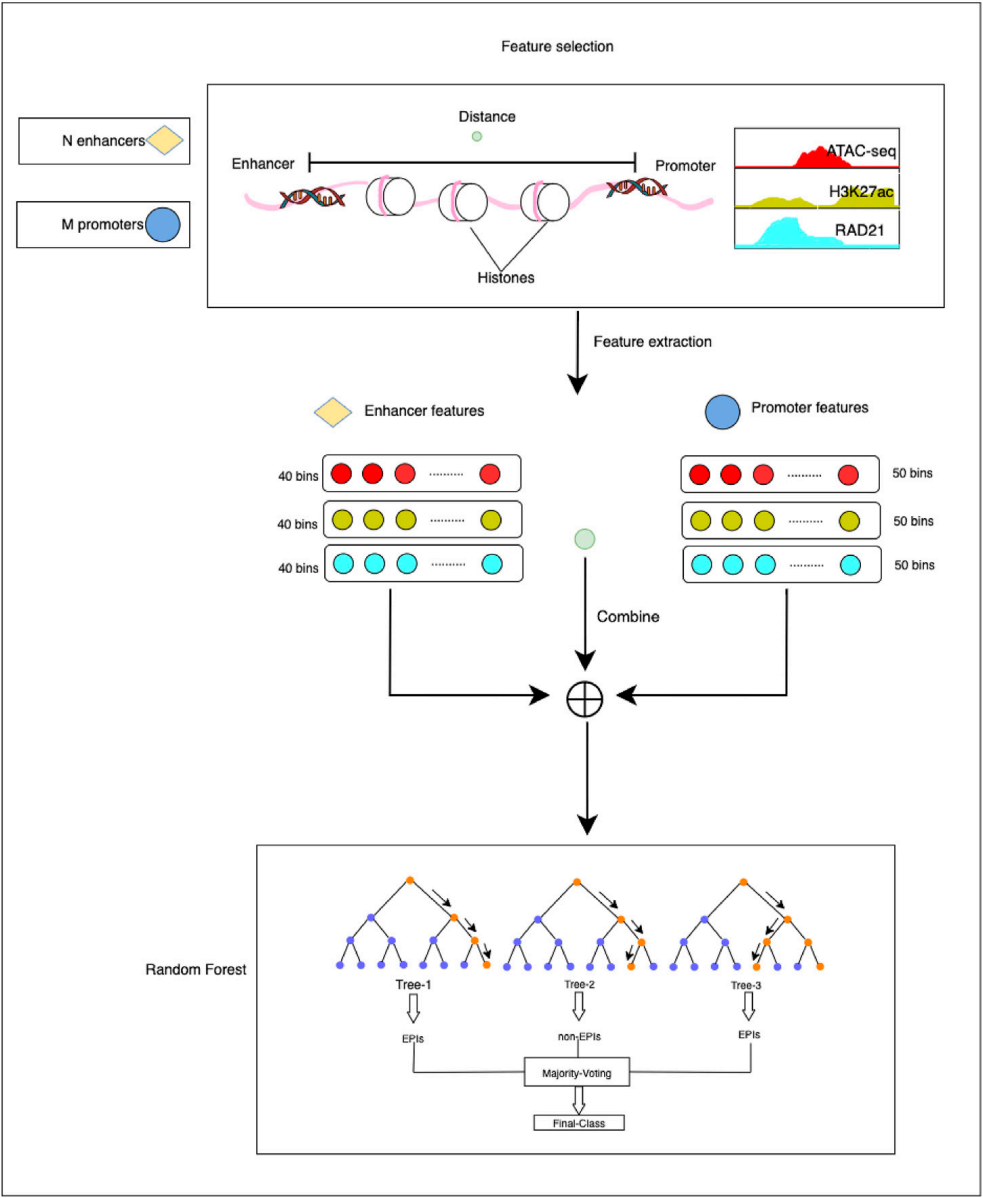
The overall framework of the HARD model. First, ATAC-seq, H3K27ac, and RAD21 epigenomic signals were selected as essential features to predict EPIs. Then, the enhancer and promoter were divided into 40 and 50 bins, respectively, with 50 bp per bin. Deeptools was used to extract the epigenomic signals. The epigenomic signal matrix was combined with the distance between the enhancer and promoter. Finally, we input the final feature matrix to the random forest learning machine for training and testing.

The HARD model consists of three primary steps: 1) constructing positive and negative sets based on the benchmark database. 2) Extracting epigenomic features that can influence the formation of EPI. 3) predicting EPIs within the same cell line and across cell lines (Figure 1).

### 2.1 Data collection and processing

The enhancer-promoter interaction data were obtained from the BENGI (Moore et al., 2020) database. To construct a benchmark of enhancer-promoter interactions, BENGI integrated various experimental datasets, such as Hi-C, ChIA-PET, genetic interactions (cis-eQTLs), and CRISPR/Cas9 perturbations. After removing ambiguous pairs, we selected the RNAPII ChIAPET data of GM12878 and HeLa cell lines with a fixed positive and negative sample ratio. Both data have a positive-to-negative sample ratio of 1:4. To ensure the data is more accurate, the ambiguous interaction pairs were removed. The RNAPII ChIAPET data only provides the IDs of cCRE-ELS (cCREs with enhancer-like signatures) and TSS (transcription start site) without the position of cCRE-ELS and TSS. We located the cCRE-ELS and TSS in the genome according to the IDs of hg19-cCREs and GENCODEv19-TSS, respectively. Then, duplicate data was removed to retain unique data.

Next, 2,000 bp upstream and 500 bp downstream of the TSS were defined as the promoter region. For enhancers, upstream 1000 bp and downstream 1000 bp were extracted from the midpoint of the cCRE-ELS region. Ultimately, 39,070 pairs of enhancer-promoter interaction were obtained in the GM12878 dataset, and 1,735 pairs of enhancer-promoter interaction were obtained in the HeLa dataset. Then, the dataset was divided into a training set and a test set for the GM12878 sample. Specifically, 80% of the data was used for training, and the remaining 20% was used as an independent test set. To ensure consistency in data distribution across both datasets, the positive and negative sample ratios of both divided datasets were maintained at a 1:4 ratio. The above data processing part and the subsequent classification experiments were implemented in the python language environment, and the sklearn library is used. The detailed data distribution is shown in Table 1.

**TABLE 1:** Distribution of samples.

Data set	Positive samples	Negative samples
**GM12878 training**	6251	25,005
**GM12878 test**	1563	6251
**Hela**	347	1388

We selected three epigenomic signal features as our experimental features, including ATAC-seq, H3K27ac, and RAD21. The epigenomic signal data, which included ATAC-seq, H3K27ac, and RAD21, were obtained from the ENCODE (Ecker et al., 2012) database. The data with IDs ENCFF000XKM, ENCFF051PGV, and ENCFF706HLO corresponded to sequencing data in bigWig format of RAD21, ATAC-seq, and H3K27ac in the HeLa cell line, respectively. Similarly, the data with IDs ENCFF000WCT, ENCFF180ZAY, and ENCFF440GZA corresponded to sequencing data in bigWig format of RAD21, ATAC-seq, and H3K27ac in the GM12878 cell line, respectively.

### 2.2 Feature extraction

The above-mentioned features were extracted through the following steps. First, the genomic site data of EPIs and epigenomic signal data were imported into deeptools (Ramírez et al., 2014), a bioinformatics tool used for feature extraction. Then the enhancer and promoter regions were divided into bins of 50 bp. Each enhancer region was further divided into 40 bins, whereas each promoter region was divided into 50 bins. For ATAC-seq, H3K27ac, and RAD21, it generated a signal value for each bin. Following feature extraction, the enhancers and promoters were represented by 120-dimensional and 150-dimensional feature vectors, respectively. The distance is defined as the number of base pairs from the midpoint of the enhancer to the midpoint of the promoter. The epigenomic feature vector and distance feature vector were concatenated to obtain the final feature matrix. This step involved combining the feature vectors obtained from the enhancer and promoter regions into a single matrix, with each row of the matrix representing a pair of enhancer-promoter interactions. The final feature matrix was then used as input for the classification experiments.

### 2.3 Classification algorithms

We compared three classifiers, random forest (RF), AdaBoost, and gradient boosting decision tree (GBDT), for predicting EPIs in the GM12878 cell line, which is considered a binary classification problem. All three classifiers proved to be efficient in solving binary classification problems.

Random forest (Breiman, 2001) is an ensemble learning algorithm. It uses multiple decision trees to classify data by randomly selecting data and feature subsets, which helps to reduce the model’s variance and overfitting risk. By voting or averaging the outputs of multiple decision trees, the model reduces the error rate and improves accuracy. In the experiment, a large amount of sample data was used, and setting the number of decision trees to 100 produced optimal performance.

AdaBoost (Schapire, 2013) assembles multiple weak classifiers to build a strong classifier, which applies to binary classification problems and has been shown to perform well on complex datasets. The algorithm assigns weights to each instance based on its difficulty level and trains weak classifiers on the weighted data. Misclassified instances have increased weight, while correctly classified instances have decreased weight. This process is repeated multiple times until the ensemble classifier reaches a satisfactory level.

Gradient boosting decision tree (Friedman, 2001) builds a model by summing multiple decision trees. It optimizes the model iteratively by adding a new decision tree that reduces the prediction error of the previous trees. The model’s accuracy improves with each iteration, making it suitable for binary classification problems. In the experiment, n_estimators, learning_rate, and subsample were set to 100, 0.1, and 1, respectively.

### 2.4 Performance evaluation

To evaluate the classification performance of the selected features and classifiers, we used six metrics: sensitivity (Sn) (Swift et al., 2020), specificity (Sp) (Swift et al., 2020), precision (Hong et al., 2020; Chen et al., 2023), accuracy (Shao et al., 2020; Yu et al., 2022), the area under the curve (AUC) (Myerson et al., 2001), and the area under the precision-recall curve (AUPRC) (Ozenne et al., 2015). These metrics serve as the basis for evaluation, and the relevant formulas for their calculation are shown below.

$$Sn=recall=TPR=\frac{TP}{TP+FN}$$ (1)

$$FPR=\frac{FP}{FP+TN}$$ (2)

$$Sp=\frac{TN}{TN+FP}$$ (3)

$$precision=\frac{TP}{TP+FP}$$ (4)

$$Acc=\frac{TP+TN}{TP+FP+TN+FN}$$ (5)

In binary classification, there are four possible outcomes: true positive (TP), false positive (FP), false negative (FN), and true negative (TN). TP corresponds to the cases where the classifier correctly predicts the positive class, while FP corresponds to the instances where the classifier incorrectly predicts the positive class. Similarly, FN refers to the cases where the classifier incorrectly predicts the negative class, and TN refers to the instances where the classifier correctly predicts the negative class. Additionally, TPR (sensitivity/recall) is the ratio of correctly identified positive instances to the actual positive instances, while FPR is the proportion of falsely identified positive instances to the actual negative instances (Zeng et al., 2020). AUC is calculated by plotting TPR against FPR at different thresholds and represents the area under the resulting curve. AUPRC is calculated by plotting precision against recall at different thresholds and represents the area under the resulting curve.

## 3 Results and discussion

### 3.1 The features of HARD model are closely related with EPI

The accessibility of chromatin structural regions is associated with the regulation of gene expression. ATAC-seq is commonly used to detect open regions of chromatin across the genome. When combined with activated histone modification, such as H3K27ac, ATAC-seq can enable the identification of specific effects on gene expression (Bravo González-Blas et al., 2019). H3K27ac is primarily enriched in enhancer and promoter regions (Herrera-Uribe et al., 2020) and is associated with gene activation (Yan et al., 2019). RAD21 and the insulator-binding protein CTCF bind to highly conserved promoters and distal enhancers, contributing to transcriptional regulation (Whalen et al., 2016; Liu et al., 2021). Numerous studies have shown that distance is a useful factor for studying EPI (Bianco et al., 2018; Al Bkhetan et al., 2019). The distance feature has an essential contribution to many models (Moore et al., 2020; Ao et al., 2022b).

**FIGURE 2:**
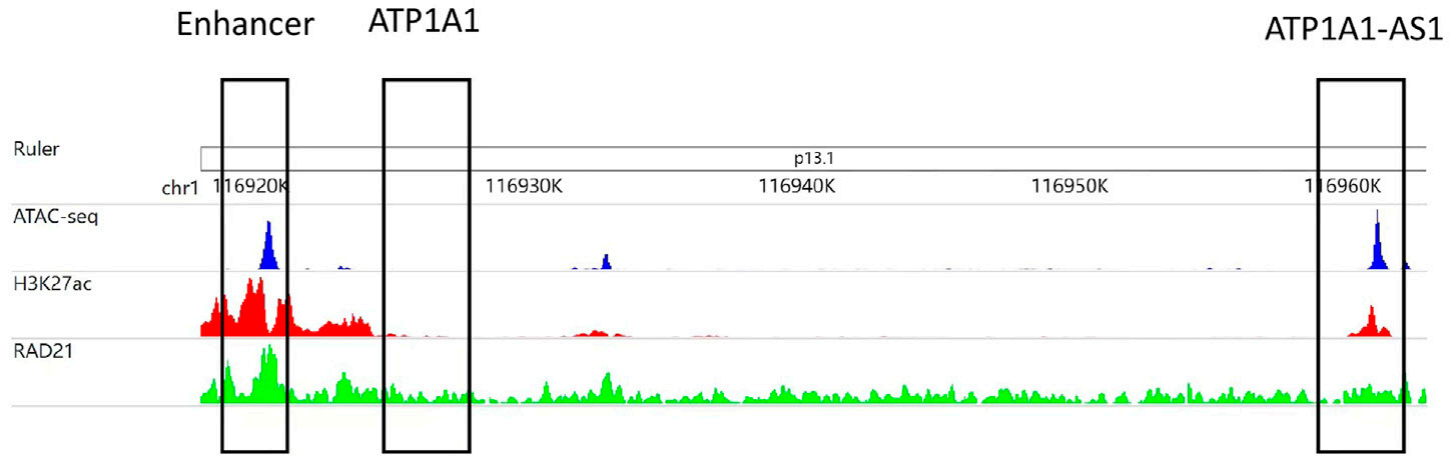
The area chr1:116,919,153–116,921,153 selected in the first matrix box is an enhancer subarea. The second matrix box selected region cr1:116,924,718–116,927,218 is the promoter region of the ATP1A1 gene. The third matrix box selected region chr1:116,959,158–116,961,658 is the promoter region of the ATP1A1-AS1 gene. The three tracks in the figure were generated from the bigWig data of ATAC-seq, H3K27ac and RAD21 of GM12878 cell lines.

Figure 2 is an example that epigenomic modification influences the formation of EPI. The enhancer region (chr1:116,919,153–116,921,153) interacts with the ATP1A1-AS1 promoter (chr1:116,959,158–116,961,658) and does not interact with the ATP1A1 promoter (chr1:116,959,158–116,961,658), according to RNAPII ChIAPET data of GM12878 cell line. In the enhancer region, the signals of ATAC-seq, H3K27ac, and RAD21 are enriched, which indicates that the enhancer is highly activated. The promoter region of ATP1A1-AS1 is enriched in ATAC-seq, H3K27ac modifications, and RAD21 binding, whereas the promoter region of ATP1A1 is not.

### 3.2 Comparison and selection of classifiers

To select the most accurate classifier, we compared three classifiers, AdaBoost, GBDT, and RF. We trained the model using 31,256 GM12878 samples with ten-fold cross-validation and evaluated its performance on an independent test set of 7,814 GM12878 samples. The classifiers were trained and tested separately, and their performance was compared using different metrics. A comparison of the metrics of the test set is shown in Table 2. Results showed that the RF algorithm outperformed both GBDT and AdaBoost in all metrics. Specifically, the RF algorithm demonstrated higher Sn, Sp, precision, accuracy, AUC, and AUPRC values, at 0.578, 0.964, 0.799, 0.887, 0.919, and 0.773, respectively. Notably, the RF algorithm displayed superior performance in AUPRC and precision metrics. The RF algorithm merges the strengths of ensemble learning and tree models, and it is capable of balancing the error for an unbalanced set of classifiers, making it a suitable choice for the dataset at hand. Consequently, the HARD model was constructed using the RF algorithm.

**TABLE 2:** Comparison of the predictive EPI performance of each classifier in the GM12878 cell line.

Classifier	Sn	Sp	Precision	Acc	AUC	AUPRC
RF	**0.578**	**0.964**	**0.799**	**0.887**	**0.919**	**0.773**
Adaboost	0.555	0.947	0.725	0.869	0.881	0.688
GBDT	0.568	0.955	0.759	0.878	0.896	0.739

The meaning of bold values is the highest value of a specific performance indicator under different classifiers.

### 3.3 Comparison with other models in GM12878 cell line

**FIGURE 3:**
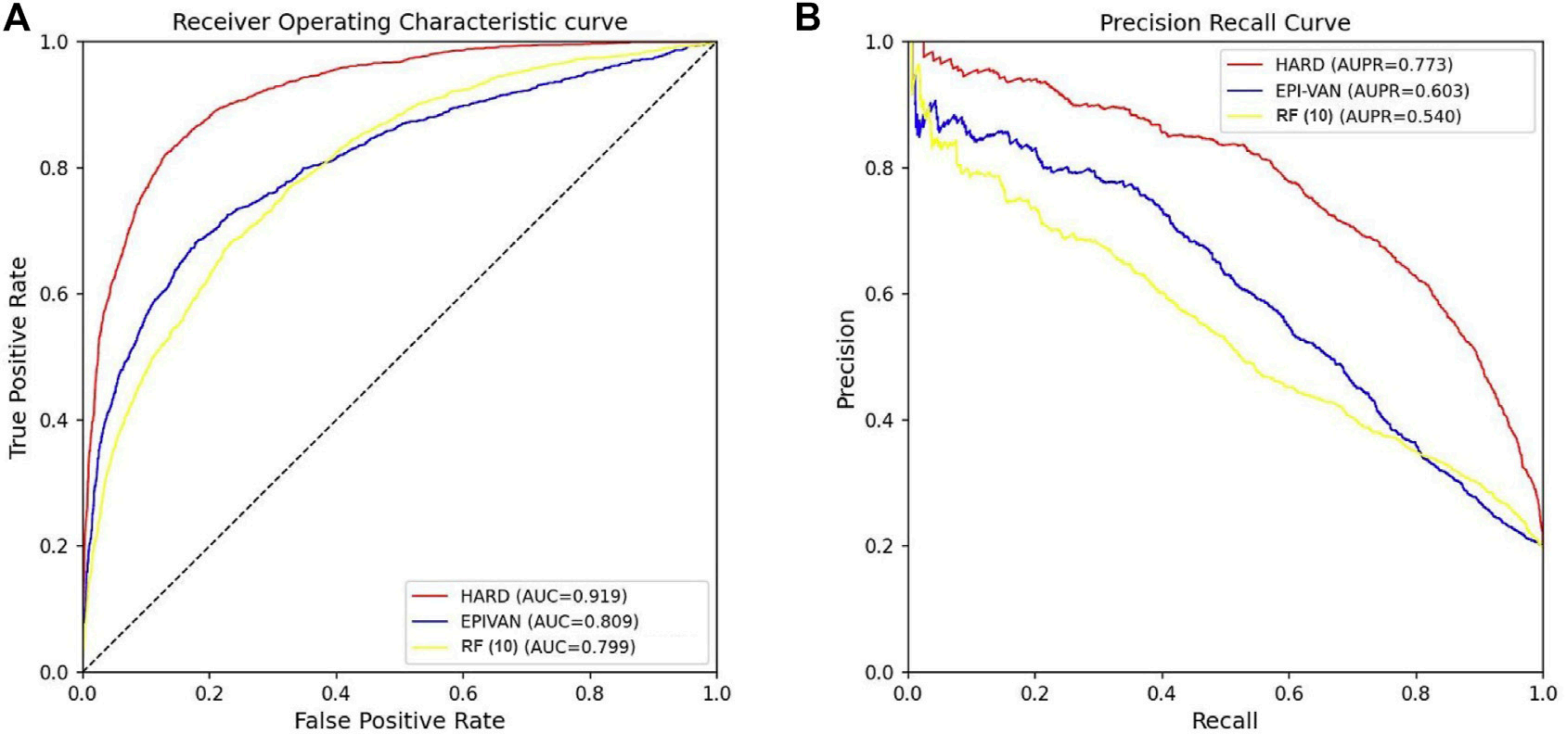
Comparison of the AUC and AUPRC performance of the three models tested independently in the GM12878 cell line. **(A)** The red curve is the ROC curve of the HARD model, the blue curve is the ROC curve of the EPIVAN model, and the yellow curve is the ROC curve of the RF (10) model; **(B)** The red curve is the PRC curve of the HARD model, the blue curve is the PRC curve of the EPIVAN model, and the yellow curve is the PRC curve of the RF (10) model.

In order to verify the validity of the HARD model, we next compared the performance of HARD against the sequence-based and other epigenomic features-based models. EPIVAN is a typical representative of sequence-based models, which outperforms the majority of existing models. RIPPLE utilizes many epigenomic features to predict EPI. These epigenomic features include cohesin (RAD21), architectural proteins (CTCF), marks associated with active gene bodies and elongation (H3K36me3, H4K20me1), activating marks of transcription (H3K4me2, H3K27ac, and H3K9ac), open chromatin (DNase I), a repressive mark (H3K27me3), and a general transcription factor (TBP). Here, we used ten available features of RIPPLE to conduct a RF classification model, named RF (10). Then the HARD model was compared with RF (10) and EPIVAN in multiple aspects. We trained the models using 31,256 GM12878 sample data with ten-fold cross-validation and evaluated them using an independent test set of 7,814 GM12878 samples. The comparison results are shown in Table 3. The results indicated that RF (10) performed best in terms of Sn, while EPIVAN produced the best results for Sp. However, each model has its strengths and weaknesses in terms of Sn and Sp. HARD had shown significant improvement in all four performance metrics compared to other models. Specifically, compared to EPIVAN, HARD shows an improvement of 7.9% and 3.7% in precision and Acc, respectively, as well as an increase of 11% and 17% in AUC and AUPRC, respectively. Compared to the RF (10), HARD shows greater improvements, with increases of 40.3%, 16.1%, 12%, and 23.3% in precision, Acc, AUC, and AUPRC, respectively. The comparison of the AUC and ROC curves of the three models is shown in Figure 3.

**TABLE 3:** Comparison of HARD, EPIVAN and RF (10) models in the GM12878 cell line.

Classifier	Sn	Sp	Precision	Acc	AUC	AUPRC
HARD	0.578	0.964	**0.799**	**0.887**	**0.919**	**0.773**
EPIVAN	0.365	**0.971**	0.720	0.850	0.809	0.603
RF (10)	**0.709**	0.730	0.396	0.726	0.799	0.540

The meaning of bold values is the highest value of a specific performance indicator under different classifiers.

### 3.4 Comparison of the HARD, EPIVAN and RF (10) model in cross-cell-lines

**FIGURE 4:**
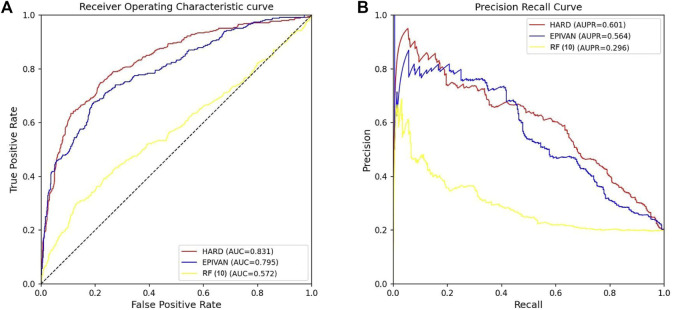
Comparison of AUC and AUPRC performance of the three models in the HeLa cell line. **(A)** The red curve is the ROC curve of the HARD model, the blue curve is the ROC curve of the EPIVAN model, and the yellow curve is the ROC curve of the RF (10) model; **(B)** The red curve is the PRC curve of the HARD model, the blue curve is the PRC curve of the EPIVAN model, and the yellow curve is the PRC curve of the RF (10) model.

To verify the robustness of the models, we conducted a cross-cell-line analysis by training the models on the GM12878 cell line and testing them on the HeLa cell line. We used 39,070 GM12878 samples as the training set for ten-fold cross-validation, and 1,735 HeLa samples as the test set for evaluation. Experiments were implemented for the HARD, EPIVAN and RF (10) models, respectively. Among the three models, HARD achieved the best performance in terms of Sp, precision, accuracy, AUC, and AUPRC, followed by EPIVAN, with RF (10) showing the worst performance. In comparison to EPIVAN, the HARD model slightly improves five metrics, only lower than EPIVAN in Sn. The HARD model outperforms RF (10) by a significant margin (Table 4). The comparison of the AUC and ROC curves of the three models is shown in Figure 4. Results indicated that HARD outperformed EPIVAN and RF (10) in predicting EPIs in cross-cell-lines.

**TABLE 4:** Comparison of HARD, EPIVAN and RF (10) models in the HeLa cell line.

Classifier	Sn	Sp	Precision	Acc	AUC	AUPRC
HARD	0.363	**0.953**	**0.660**	**0.836**	**0.831**	**0.601**
EPIVAN	**0.513**	0.890	0.539	0.815	0.795	0.564
RF (10)	0.144	0.949	0.402	0.786	0.572	0.296

The meaning of bold values is the highest value of a specific performance indicator under different classifiers.

## 4 Conclusion

The interaction between enhancer and promoter is a complex process. Various genomic and epigenomic features are related to EPI. Many machine learning models have been developed to predict EPI based on a large number of genomic and epigenomic features. The redundancy of features leads to unsatisfactory experimental results and limits the application to more cell lines. In this paper, we developed the HARD model, which employed a minimal number of epigenomic features to predict cell-line-specific EPIs. It is noteworthy that the HARD model is based on benchmark data from the BENGI database, which defined EPI strictly by integrating ChIA-PET, genetic interactions (cis-eQTLs), and CRISPR/Cas9 perturbations. By comparing with two other models, we found HARD outperformed them both in the same cell line and cross-cell-lines. Importantly, our model only used H3K27ac, ATAC-seq, RAD21, and Distance as input, which makes it possible to apply to more cell lines.

## Data Availability

The original contributions presented in the study are included in the article/Supplementary Material, further inquiries can be directed to the corresponding author.
